# Are therapeutic communities therapeutic for women?

**DOI:** 10.1186/1747-597X-1-3

**Published:** 2006-01-12

**Authors:** Michele J Eliason

**Affiliations:** 1 College of Nursing, The University of Iowa and Institute for Health and Aging, University of California, San Francisco, USA

## Abstract

This paper addresses the growing phenomena of therapeutic community (TC) treatment approaches for women in correctional settings. Although rapidly increasing in number across the country, there is very little empirical research to support the effectiveness of TC treatment for women. Therefore, the literature on the efficacy and effectiveness of TC treatment for women is reviewed in relation to the literature on women's treatment issues. The literature review highlights the gaps where TC treatment ignores or exacerbates issues that are common to addicted women, or uses methods that may be contradictory to women's recovery.

## Introduction

In the past ten years, there has been an explosion of new therapeutic community (TC) programs in prisons across the United States. By 2001, nearly every state in the country had at least one TC program in a prison within its borders, or was planning to start one, and 31 states had a TC program for women [1]. Like other types of substance abuse or criminal thinking programs/approaches used in prisons, TCs have been put in place in women's correctional settings without regard to whether they are the best approach for women. Under the guise of gender-neutrality, programming developed for and tested on men has too often been uncritically applied to women. This paper will review the theory and research supporting the use of TCs in relation to the literature on gender-specific issues in substance abuse treatment. The core philosophy, principles, and tools of a TC will be examined given what we know about women's development and treatment needs.

### Research on the female offender

The growing body of research on the differences between male and female substance-involved offenders strongly suggests the need for gender-specific services. This research has identified different antecedents and consequences to abuse, different patterns of drug use, different barriers to treatment, and different relapse triggers. In addition, the types of crime and motives for criminal activity differ by gender. Some of the major differences are summarized below. A much fuller account of this literature can be found in a comprehensive report commissioned by the National Institute of Corrections [2].

#### Physical and sexual abuse histories

According to Bureau of Justice Statistics data, 40% of incarcerated women compared to only 9% of incarcerated men report a lifetime history of abuse. These figures are based on prison intake records that may underestimate abuse experiences. Other studies report much higher figures for women. For example, some studies have found rates of childhood abuse as high as 70–80% [3-5]. There is a considerable literature that links early trauma and abuse histories to adult substance abuse as women learn to self-medicate the emotional distress created by abuse [6]. In fact, childhood abuse experiences are the primary pathway to substance abuse and incarceration for women. Even when women and men report similar rates of trauma and abuse, there may be differences in the experiences of abuse. For example, rates of physical abuse may be similar in women and men, but rates of sexual abuse are higher in women. Sexual abuse is much more personal, affecting women in the most sensitive aspect of their development – their sexuality – and the perpetrators are often the persons who are supposed to love and protect the child. Early victimization experiences often create emotional distress and/or post-traumatic stress symptoms or disorder, leading to self-medication of the negative emotional states. If substance abuse treatment does not address unresolved underlying trauma and abuse issues and replace self-medication with healthier coping mechanisms, then treatment is unlikely to be successful. Clinicians who are aware of trauma and abuse issues can have significant impact on their client's outcomes, and growing evidence that substance abuse treatment is not as effective unless the trauma issues are treated concurrently [7-9]. Creating trauma-informed treatment may facilitate recovery, but prisons are not the most conducive environments for trauma reduction efforts.

#### Substance use/abuse patterns

More women than men in the criminal justice system report substance abuse and dependency, with 80% of women compared to 50% of men reporting recent substance abuse [10]. In most studies, women tend to report much greater drug use and negative consequences of use whereas men report greater alcohol use. In Iowa, women were much more likely to report stimulant drug abuse (methamphetamine and cocaine accounted for two-thirds of the drugs of choice) whereas men reported alcohol and marijuana use much more often [11]. People may choose substances that balance their emotional state – thus depressed women may select stimulants to feel some semblance of normalcy. Stimulants may also be preferred by women because of their role in weight loss. The treatment approaches do not differ substantially depending on the substance [12], but relapse triggers may be different.

#### Criminal behaviors and pathways

The vast majority of women committed crimes after their initiation to substance abuse and dependency, and their crimes appear to be much more likely to be motivated by economic need than men [13,14]. Drug use is the major pathway to crime for women [10,15]. The majority of women are incarcerated for nonviolent offenses and pose little or no danger to society. Even when they have committed violent crimes, they are often directed to intimate partners who inflicted years of abuse upon them. The literature does not support the existence of the "career criminal, antisocial" type among many women. In most cases, crime was motivated by economic need, drug addiction, and/or their intimate relationships.

#### Physical and mental health

Women appear to suffer negative consequences (both physical and mental) from substance abuse earlier than men. This "telescoped" development is revealed as more alcohol and drug related physical problems and more mental health problems as antecedent and consequence of drug use [14,16,17]. Women also have reproductive health problems that do not exist in men. About 6% are pregnant while in prison, and many have high-risk pregnancies because of their chronic drug use [18]. Incarcerated women are 50% more likely than men to be HIV positive and have a wide range of sexually transmitted infections [19]. At least one-fourth of incarcerated women have a diagnosed mental disorder, the most common of which are depression and Post-Traumatic Stress Disorder. These often co-exist with substance abuse [20,21]. Jordan et al. [4] reported that 64% of female offenders in their study had a lifetime history of mental illness, and that 46% had experienced symptoms in the past six months.

#### Marital, intimate, and family relationships

Women in the criminal justice system have different patterns of relationships than women in the general population and are much more likely to have never married (46% have always been single). Less than 20% are currently married and about one-third are divorced or separated [10]. However, 70% have at least one minor aged child and most women retain custody of their children while incarcerated. When a father is incarcerated, about 90% of the time the child's mother will be the caretaker, causing little disruption in the child's life [22]. However, because of the marital/partner status of drug-addicted women, their children must often be placed with parents, other relatives, or placed in the foster care system. Even though nearly half of women never receive a visit from their child while incarcerated (usually due to transportation problems), they often remain very involved in their children's lives [23].

Spouse and partner relationships may be a contributing factor both to women's initiation of substance use and their involvement in crime. Many women are introduced to illegal drugs and/or drug-dealing through male intimate partners [24,25]. A considerable number of incarcerated women experienced domestic violence in their adult relationships [26], and if these issues are not dealt with in treatment, they often return to the abusive partner and resume substance use and abuse.

Another way that women in criminal justice settings differ from men is that they are more likely to have consensual intimate and/or sexual relationships with other women. These relationships can mimic the dysfunctional relationships they had outside of prison, or they can sometimes represent the first healthy relationship of the woman's life [27]. These relationships can create confusion for women who have always thought of themselves as heterosexual. Intimate/sexual relationships in early recovery are discouraged or forbidden in many types of treatment approaches, but particularly in TC programs where they are seen as disruptive to the development of the overall community.

#### Education and employment

While women frequently report educational levels similar to incarcerated men, they often show large differences in employment status and income levels. According to BJS data [10], 40% of women compared to 60% of men were employed full time prior to arrest, and 37% of women compared to 28% of men had incomes of less than $600 per month. Coupled with their greater responsibilities for children, more women live in poverty than men. However, vocational training opportunities for incarcerated women are less available than for men [28].

#### Treatment engagement

Women differ from men in several ways, many of which may influence their motivation to engage in treatment and their response to treatment. On the one hand, women may be more motivated to engage in a TC type of treatment because women are socialized to communicate about emotions and to seek help and support from others. Sharing emotional intimacy may be somewhat easier for women than for men. In addition, the majority of women in prison are mothers [10] and the desire to become better parents and re-unite with their children may be a strong motivator for treatment. On the other hand, women enter prison with fewer job skills and opportunities than men and commit more crimes for economic survival reasons than men. Women's family caregiving responsibilities may hamper their ability to get job training or higher education that would improve their employment possibilities, increasing the chance that they will return to crime to make ends meet. Prison rarely provides remedies for these problems, so women re-enter the community and often resume their family caregiving activities with the same low job skills they had before prison, with the added stigma of being an ex-convict, making it even harder to find gainful employment [29-32]. In some prisons, entering a TC program may interfere with the ability to participate in job training or employment activities. Many women will choose jobs over treatment as a matter of economic survival. Because conviction of drug felonies will render many women unable to access food stamps, welfare benefits, Section 8 housing, and Pell grants, job training becomes even more critical.

### The case for gender-specific treatment

The research literature reviewed above is merely the tip of the iceberg of data on female offenders, and it is increasingly clear that the significant differences between substance abusing women and men warrant consideration of gender-specific treatments. Men often benefit from co-ed treatments because of women's socialization as the caretakers of the culture – women in treatment will often sacrifice their own recovery to take care of others. In addition, treatments that have been developed for men often do not address the major treatment issues of women, such as parenting, relationships, trauma and abuse, developing a sense of self, and sexuality.

In conclusion, there is a growing support that women could benefit from gender-specific treatment whether the woman receives TC or other forms of treatment [33-35]. What is not yet clear is whether TCs, as currently conceptualized, are the best model for delivering gender specific treatment.

### What are TCs?

In recent years, there has been renewed concern about the "revolving door" of prison, and strategies to facilitate offender reentry to their communities have been proposed [36-38]. Providing rehabilitative services while in prison has been proposed as one possible solution, and substance abuse treatment is clearly a high priority need [38]. Drugs are the major reason for the initial arrest of women [10], the increase in incarceration of women [39], and the major reason for parole violations [17], although women play "no substantial role in drug trafficking" [[40], p.3). Prison and other correctional administrators have long recognized the need for drug and alcohol treatment, and a variety of different types of programs have been developed. Until recently, these programs were often cognitive-behavioral in nature, focusing on the intersection of criminal thinking and substance abuse that is thought to be common to the "antisocial personality disorder" male offender. That is, programs were tailored to the profile of the male offender.

In the late 1980s, therapeutic community (TC) programs began to emerge in a few prisons. They were fairly common in communities and in psychiatric units in the 1960s, but as lengths of stay decreased because of changes in payment systems, most communities could no longer sustain these lengthy programs. Prisons, on the other hand, were ideal places to institute comprehensive, long-term substance abuse treatment programs. TCs were hailed as not only a way to treat substance abuse, but also to introduce prosocial values that reduce criminal thinking and activity. Now, nearly every state in the United States has at least one prison-based TC program, and many states have several.

Therapeutic communities strive to develop closely knit groups of supportive people. This is achieved by participating in a highly structured, rigid environment with a specific lingo and ritualized procedures extending for a period of several months to more than a year. Although prison-based TCs vary considerably from one program to another, they have some shared features: they are based primarily on a social learning model; participants are isolated from the influence of the prison's general population; participants experience a total immersion into treatment where everyday activities become part of the treatment regime; and peer counseling and support is a major part of the therapeutic intervention [41,42]. Theoretically, the community itself is the primary therapist [43]. The U.S. Office of National Drug Control Policy [44] commissioned standards for prison-based TCs, establishing the core principles and approaches of a TC, and outlining four major philosophical issues for all prison-based TCs :

1. Substance abuse and criminality are symptoms of a disorder of the whole person.

2. The disorder of the person consists of social and psychological characteristics that must be changed.

3. "Right living" refers to the morals and values which sustain recovery, and is the goal of treatment.

4. Recovery is a developmental learning process.

In a TC, addiction is not viewed as a sickness or disease, but rather as a learned condition that can be changed. This is accomplished in a "continuous (24 hour) atmosphere of constructive confrontation and feedback" where "the culture is defined by a mutual self-help attitude where community members confront each others' negative behavior and attitudes and establish an open, trusting, and safe environment where personal disclosure is encouraged and the prison culture in the general population is rejected" [[44], p. 3]. While confrontation is a basic part of the treatment, practices that are demeaning or humiliating are prohibited.

The typical TC operates via a morning meeting and evening meeting of all residents; a hierarchical chain of communication where every member has responsibilities and is accountable to someone; an elaborate system of accountability including verbal and written pull-ups (notification of negative behaviors) and push-ups (positive acknowledgements). Failure to address negative behaviors in a timely fashion can result in community-wide confrontation groups ("hair-cuts").

### Outcome research with men

Studies with men have shown that TCs can have impressive effects on recidivism rates. Several studies have demonstrated a greater than 50% reduction in recidivism [45-48]. Knight et al. [46] reported these differences in recidivism over a three year period: of ex-offenders with no treatment, 42% recidivated; ex-offenders who attended a TC unit but had no community aftercare had a 64% recidivism rate; and TC graduates with community aftercare had a recidivism rate of 25%. If only commission of new crimes was considered, the recidivism rate of the TC plus aftercare group was 4%. TC treatments with an aftercare component have been found to be equivalent to or superior to other forms of prison-based substance abuse treatment that generally result in recidivism rates of about 40% [49]. When outcomes beyond recidivism are examined, TC treatment for men has been associated with reductions in drug use, decreased income from crime, fewer hospitalizations, and increased likelihood of having health insurance [45]. The research of the past ten years exploring outcomes for men up to three years after release from prison has consistently shown positive effects of TC programs in prison if community aftercare programs are also included. However, there are some limitations to this body of evidence: many of the studies did not use treatment control groups, or they compared TC programs to shorter, less intensive programs, and there are few randomized clinical trials (the gold standard of treatment outcome research).

### Research on outcomes for women

Wexler et al. [50] examined outcomes from 247 women in a New York TC. These women had a lower re-arrest rate than control group women who participated in other types of rehabilitative programs (18% compared to 29%). Jarman [51] compared 196 women in a California prison TC to women who did not participate in TC programming, but did not find differences in success on parole. In another evaluation of the same program [52] with a smaller sample of 47 TC grads and 49 wait list controls, more of the TC women were successfully discharged from parole. The TC women also reported lower rates of use of heroin and amphetamines at follow-up, but used more marijuana and cocaine than the control group women. A study from the Federal Bureau of Prisons suggested that of 547 women studied, TC treatment was not effective in reducing recidivism or relapse [53].

Inciardi [54] reported that women completing a therapeutic community program in a Delaware prison were more likely to abstain from drugs (65%) than control women (30%), and were less likely to be re-arrested (18% compared to 38%). Messina et al. [55] compared outcomes by gender for women and men assigned to TC treatment plus community aftercare. On admission, the men were more likely to have a diagnosis of antisocial personality than the women, and the women were more likely to have a diagnosis of depression than the men. In addition, the women were much more likely to report a history of physical and sexual abuse and these experiences affected substance use relapses for women, but not men [25,56]. This finding suggests that in women's TC units, abuse experiences must be addressed directly and treated as a major relapse trigger.

Farrell [57] compared 41 women who completed a TC program and were currently in a transitional TC in a community setting, to 37 women in work release programs. The TC group showed a lower relapse rate for alcohol, but not for other drugs. Women in the TC group were more successful at forging some sorts of social support systems in their communities after release from prison, but were not more likely to be taking care of children, holding down jobs, or having significant relationships outside of their TC support network. Women in both groups reported that they felt disconnected from their larger communities. There was no difference in the recidivism rate by group, although having responsibility for children was a significant predictor for women in either group for staying out of trouble. No information was provided as to whether the TC program provided gender-specific programming such as attention to early life abusive experiences or parenting.

Schinka et al. [58] reported on changes in mood and personality characteristics in women one year after admission to a TC program, finding that the women reported fewer depressive symptoms, and a decrease in avoidant, dependent, self-defeating, and borderline personality characteristics. TC treatment had no effect on signs of antisocial disorder.

In conclusion, the research on the effectiveness of TC programming for women is scant, and the few outcome studies available provide mixed findings [59]. One reason for the difference in outcome data for women and men might be that the programs are not directly comparable. There appear to be philosophical differences as well as variations in treatment approaches, emphasis, and topics of meetings in women's and men's TC programs. However, in spite of the lack of evidence, prisons and community correctional settings across the country continue to introduce new TC programs for women.

### Are TCs likely to meet female offenders needs?

Why are the outcome studies of the effects of TCs equivocal for women when they are overwhelmingly positive for men? There may be characteristics of TCs that are much more compatible with the needs of men found in prisons than with the needs of women who are incarcerated. Men are much more likely to have a diagnosis of antisocial personality disorder whereas women are more likely to have mood and anxiety disorders and/or borderline personality disorder. The highly confrontational, closely supervised, and "in your face" nature of TC programming may provided clear cut boundaries and structure for antisocial men, but trigger PTSD symptoms and contribute to feelings of helplessness and low self-esteem in depressed women.

Women are socialized to communicate indirectly to avoid hurting another person's feelings, and women often take criticism as a reflection of their self-worth. Thus, the direct communication of the TC, which is often very public in nature (when the unit coordinator reads written pull-ups out loud in meetings for example), can be devastating to some women.

TCs also give mixed messages about relationships. On the one hand, women are told to be open and vulnerable to their community, but not to form strong bonds with one or a few others. Outside of prison, women's friendship networks tend to include one or just a few intimate close friends, not a unit of 25 or 50 women. When staff notice close relationships forming between women, they are quickly labeled as pathological and steps are often taken to separate the women.

There is a fine line between teaching accountability, blaming the victim, and using guilt and shame to motivate behavior. Many women report that TC tools such as pull-ups are used to retaliate or to intimidate women, who then have no recourse or ability to share their side. In the hands of skills and mature individuals, the TC tools can be powerful calls for accountability and acknowledging one's negative behaviors can be helpful, but when used inappropriately, by peers early in recovery, these tools can be detrimental to recovery.

## Conclusion

Therapeutic Communities were instituted in prisons for all the right reasons – there was a desperate need for effective substance abuse treatment programs and TC's were well-suited to the prison environment in many ways. The format and content of TCs was developed to address the antisocial man, and it appears that TC treatment is fairly effective with this population. However, women's socialization and the adverse consequences of trauma and abuse that most incarcerated women have experienced make the TC a potentially toxic environment for many women. It is certainly possible to modify the TC model to soften its confrontational nature and address gender-specific issues, but then is it still a TC? It may be preferable to develop programs specifically for women rather than to continue to apply and later modify, programs developed for men.

## Competing interests

The author(s) declare that they have no competing interests.

## Authors' contributions

The author declares that she is the sole author of the article.
